# “*I Guess When You Start to Feel the Suffering Again it Becomes Unbearable”*: Mapping the Phenomenon of Loss of Compassion and Empathy, and Rise of Cynicism in Medical Trainees

**DOI:** 10.1007/s40670-026-02763-y

**Published:** 2026-06-20

**Authors:** Beatrice Preti, Leenah Abojaib, Joshua Grief

**Affiliations:** 1 Department of Oncology, Western University, London, ON Canada; 2 Department of Haematology & Medical Oncology, Emory University, Atlanta, GA USA; 3 School of Health Professions Education, Maastricht University, Maastricht, Netherlands; 4 Emory School of Medicine, Emory University, Atlanta, GA USA; 5 Michael G. DeGroote School of Medicine, McMaster University, Hamilton, ON Canada

**Keywords:** Medical students, Residents, Physicians, Compassion, Empathy, Cynicism

## Abstract

As medical learners progress through their training, compassion and empathy decrease, while cynicism increases. Though factors contributing to this evolution, such as fatigue and workload, have been identified, the process through which these changes occur has not been studied in detail. Our goal was to explore this process, mapping out key points in the transition which might be targetable for educators and leaders. Consequently, we conducted a qualitative study in the qualitative descriptive approach, analysing the process through which compassion and empathy decrease and cynicism increases over time. First, we collected Reddit narratives to develop an understanding of the process from a variety of perspectives. Subsequently, we invited participants (medical student to attending physician) to provide detailed descriptions of the process. 77 Reddit narratives and eight participant descriptions comprised 85 data slices. Analysis of the data identified four steps through which compassion and empathy are generally lost, and cynicism increased. Steps consisted of a real-world awakening to the realities of the medical field, interpersonal struggles compounded by lack of support, realised emotions in response to these realities and struggles, and development of a coping mechanism. While this process is not a unidirectional, linear one, these four steps help to describe how the evolution from compassionate, empathetic junior learner to more cynical physician occurs. This offers several concrete potential points of intervention and study for future work.

## Introduction

The term “compassion” has historical roots and a variety of definitions; in this study, compassion is defined as a response to the suffering of another, consisting of an urge to relieve said suffering [1]. In the medical field, compassion is intertwined with the concept of “empathy” [2]; empathy entails more simply seeing and understanding the perspectives of another [2], without personal involvement. Despite differences in the conceptualisation of compassion and empathy, including whether the two constructs can exist as separate entities or are intrinsically connected, compassion and empathy are acknowledged as desirable in medical professionals, to the point where curriculums exist to teach these traits [3]. For the purpose of this study, compassion and empathy are considered separate, though linked, concepts.

Published literature suggests that, as medical learners progress through their training, compassion and empathy decrease [4, 5]. During the same time period, medical learners have been noted to develop increasing cynicism [6]. While cynicism, likewise, has a variety of context-depending definitions, in the medical education field, cynicism is described as an indifference, doubt, or even contempt for the suffering and situations of others [7].

The medical profession is known to be a demanding one – and its training process is no exception. Loss of compassion is associated with stress-related conditions, including burnout [8] and compromised performance [9]. However, these are vague associations without details, highlighting the importance of better understanding the process, with a lens to identifying concrete points for intervention. While the transformation from compassionate, empathetic medical learner to cynical junior physician is well-documented in literature spanning over 100 years [10], the specifics of how this process unfolds is not.

Consequently, we conducted a study exploring the process of loss of compassion and empathy, and rise of cynicism, in medical trainees. Our goal was to highlight stages or phases in the process in order to offer potential intervention points for educators and researchers [11]. 

## Methods

A qualitative methodology was selected to allow for a more comprehensive exploration and understanding of each individual participant’s experience and thoughts, thus allowing for the most meaningful data collection possible [12]. Specifically, qualitative description was selected due to its suitability to address the research goal while aligning with the proposed research paradigm [13]. This methodology allows for description of a phenomenon to generate a general understanding of the process through which empathy and compassion are lost and cynicism is increased. This study adopts a relativist ontology (multiple truths can exist), subjectivist epistemology (understanding is created during participant-researcher interactions), and a constructivist paradigm (understanding is co-created by participants and researchers).

Of note, while initial versions of the research goal contained reference to “joy”, this was not found to be a major factor in descriptions or analysis, and has been removed from the results and discussion. However, given the debate around whether compassion and empathy are separate, linked, or two facets of the same concept [11], we include both of these terms for completeness.

In order to obtain a robust understanding from multiple angles, two different data sources were used to provide cross-sectional “slices” of perspective and reference [14]:

- Reddit Narratives: Social media posts have previously been used to gain insight into the learner experience [15, 16], including the loss of compassion specifically [17]. We collected Reddit posts from the *r/medicalschool* and *r/residency* Reddit communities (subreddits) containing narratives on the topic of losing compassion and empathy or developing cynicism. Search terms consisted of: *(“student” OR “resident” OR “fellow”)* AND (“compassion” OR “joy” OR “empathy” OR cynic*) published between June 1st 2023 and May 31st 2024. We included posts written in English, and excluded posts written by an individual identified as not involved directly in the medical education journey (i.e. nurses, allied health students). Medical students, residents, fellows, and attendings were all included, as their stories and perspectives represented different stages of the medical education journey, and we were interested to see the evolution over time. Posts were collected and examined by authors LA and JG; inclusion in the analysis was based on consensus.
  - Specific inclusion criteria were: posts written in English, comprised either a main post or a comment, and contained a narrative depicting the loss of empathy/compassion or rise of cynicism. Narratives were defined as stories with a clear beginning, middle, and end.
  - Specific exclusion criteria were: posts written by a non-physician/non-physician trainee, contained hearsay (not a first-person account), and/or clearly satirical.
- Participant Responses: After the Reddit phase was complete, individuals involved in the medical education process (again, students to teaching faculty) were invited through emails to key informants, social media posting, and snowballing to participate in a detailed written reflection or unstructured interview, in response to a single prompt: “Previously-published studies have noted a decrease in compassion, empathy, and joy in medical trainees over time, and a rise in cynicism. Can you please describe a situation or situations that this statement reminds you of?” This prompt was used as a consolidation for the findings from the Reddit narrative portion of the study, which overwhelmingly supported previous research exploring the loss of compassion/empathy and rise of cynicism. The use of this prompt allowed a deeper understanding of the themes collected in the Reddit phase of the study, and helped to overcome some of the limitations of Reddit/narrative-only studies (including concerns about transparency/authenticity in Internet repositories). In line with unstructured interviews, further questions/elicitation were decided in the moment based on the direction of conversation. Interviews occurred virtually using Microsoft Teams, and lasted 30–60 min. The participant response period occurred from January – July 2025.

**Fig. 1:**
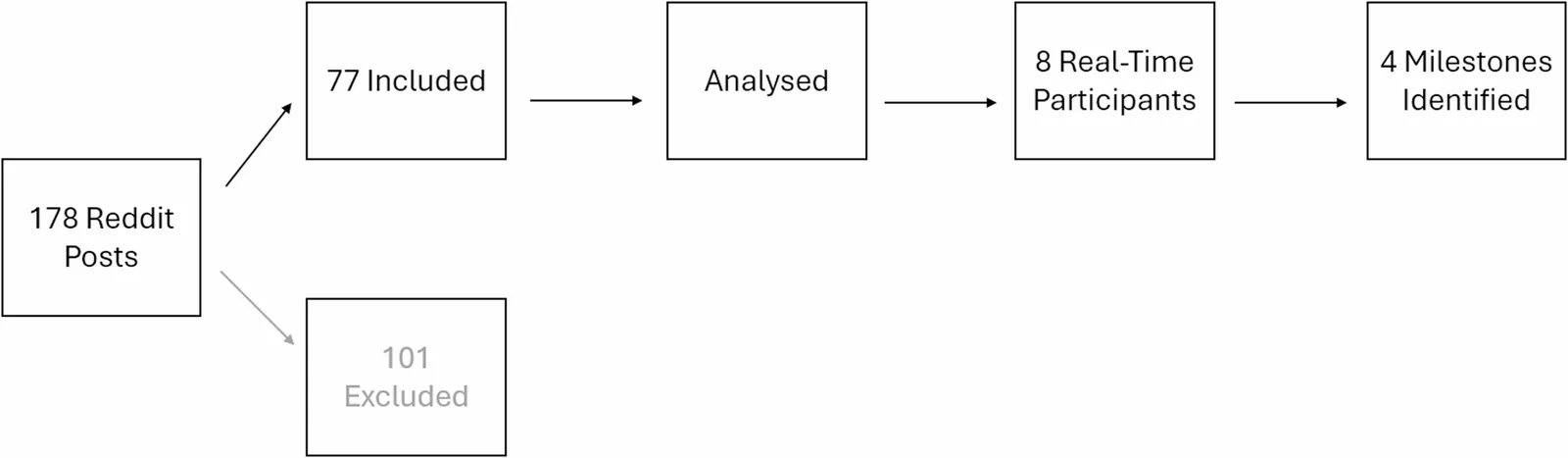
Study design

Figure 1 below provides a schematic of study design.

In accordance with qualitative description, analysis was performed through content analysis [13]. Narratives and transcripts were inductively coded by author BP using in vivo and descriptive coding [18] conducted in Microsoft Word; codes were then condensed into categories until the four framework steps were established. Steps were discussed amongst the author team, and a consensus was reached prior to finalising the framework presented in the results. Data collection was terminated at the point of theoretical saturation [14], where the addition of more information did not add new insights to the data analysis. This was achieved after five participants, although three further participants were included for quality assurance.

Data was de-identified and stored on a secure university-based OneDrive. Participants were asked to avoid using genders or proper nouns (including names) to assist with anonymity.

This study was conducted as part of the International Association of Medical Science Educators (IAMSE) Medical Educator Fellowship programme. As such, regular discussions and debriefing with peers and mentors about study design, methods, and results were held throughout the study process, and feedback was incorporated.

This study received Research Ethics Board approval from Western University. Of note, Reddit is an open public Internet repository of narratives, and consent from authors who have already published freely on the Internet was not required. Written consent was obtained from participants in the second part of the study.

## Results

A total of 77 Reddit narrative posts (178 screened, 101 excluded based on inclusion/exclusion criteria) and eight participants were included in this study. While Reddit does not require geographic location disclosure, terminology suggested a largely North American audience. A total of 8 participants involved in Canadian medical education (3 students, 2 residents, 3 consultants) participated. Three participants chose to participate in written reflections (2 students, 1 resident), whereas five participated in interviews. Through analysis of cross-sectional narratives from individuals at a variety of levels of training (medical student to teaching attending), we identified a four milestones through which compassion and empathy are lost, and cynicism is developed, delineating clear stages (albeit with potential temporal variation) in the process. These include a wake-up to real-world medicine, interpersonal struggles, realised emotions, and finally finding a way to cope through decreasing compassion and empathy while increasing cynicism. It is important to note that these represent non-linear, key experiential milestones with a great deal of overlap, rather than a unidirectional, unilateral process.

**Fig. 2:**
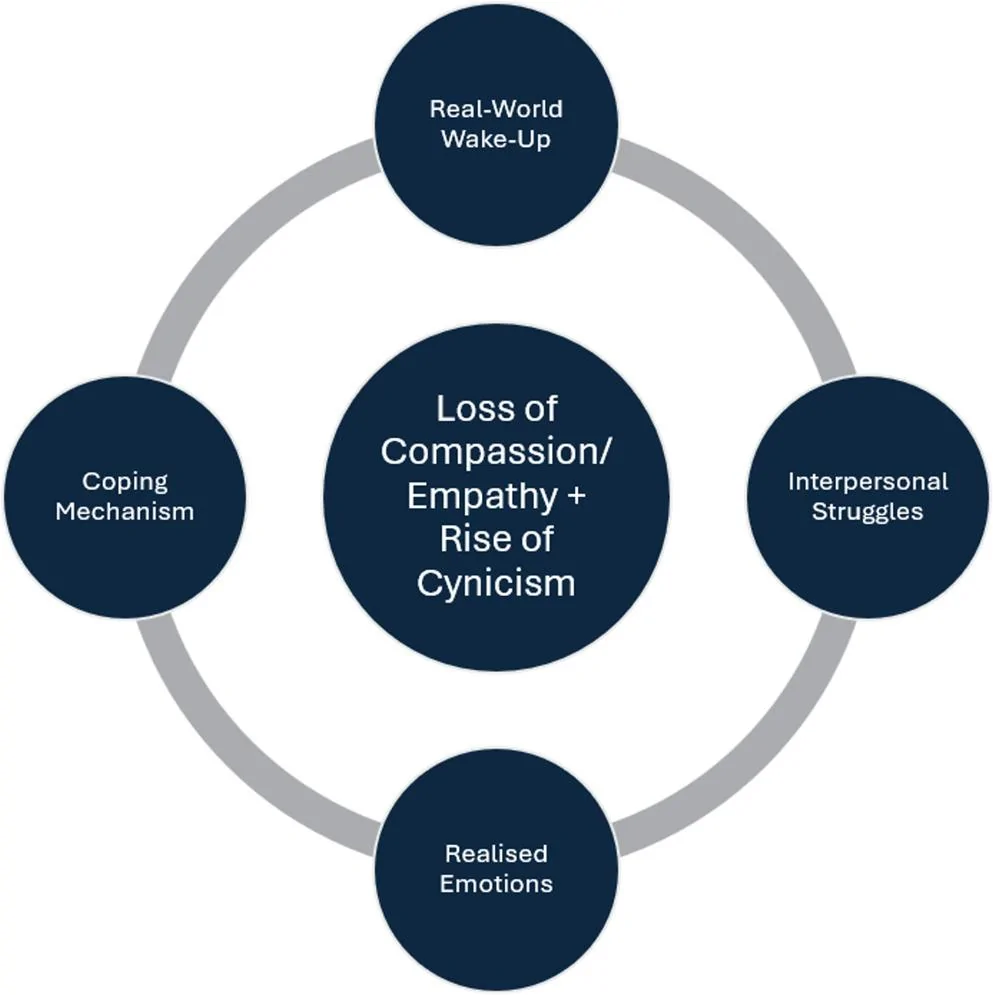
Visual representation of study results

A visual representation of study results is presented in Fig. 2.

### Real-World Wake-Up

Participants, by and large, reported feeling caught “off guard” by the realities of medicine. They reported discomfort as they learnt about their new field, finding that previously-held values and beliefs around healthcare and medicine often don’t play out in the real world.

*“…the way that they just talked about the patient in front of the patient I thought was interesting*,* and I don’t know if it’s like the culture of the ICU or because I’m all new to it. But that was something that made me uncomfortable”* (P2).

These realisations led to doubt about decisions made to pursue a medical career, disillusionment with the healthcare system, feelings of institutional betrayal, and feeling like a cog in the wheel of an immoral system. Some participants cited an unexpected moral dissonance, such as conflicting pressures from higher-ups or impossible choices in patient care:

*“I have definitely become more “jaded” throughout the two years of my clinical rotations given the negative experiences with patients*,* pressure from my own teams*,* and the frustrating reality of our healthcare system”* (P6).

Some participants cited tension when they realised a lack of meaning or lack of results of their efforts, which differed from pre-medicine life, where efforts in, for example, studying diligently translated to high marks. Along those lines, many participants mourned the loss of their pre-medicine life.

*“Undergrad was amazing. I had a blast…enjoyed friends and parties and dreaming big about the future.*

Med school…was an absolute time suck…Residency is absolutely f***ing detestable. I work 90 hours a week,* q3/q4 26 hour call and i hate waking up in the hospital every time I am on call IF i get the chance to nap at night. I want to just obb* [sic] *the building when I wake up and find myself there.”* (Reddit Post).

They also noted the impact their career had on their relationships and personal life:

*“…not being able to bring your best self home to your children and spouse when you’ve given all your compassion to people you will never see again”* (P1).

Participants, particularly early-stage trainees, noted fears of losing compassion and attempts to preserve it. They also noted consciously realising the loss of their compassion and the slow creep of nihilistic attitudes towards their work, and tried to work against it. More seasoned participants, however, noted that the process seems inevitable and even necessary (see 4th stage).

## Interpersonal Struggles

As they progressed through their training, participants noted struggles and frustrations with others.

One of the most prominent struggles was with patients, including clashes with patient attitude, mindset, and behaviour. Participants noted that repeated encounters with certain patient behaviours (such as lack of follow-through of medical recommendations or aggressive behaviour towards healthcare providers) decreased their interest and empathy in patients exhibiting similar behaviours, or even patients in general. This led to dehumanisation of patients and/or conscious separation of emotions from the situation. In some cases, such patient behaviour was described as “abuse”; in other cases, lack of efforts from patients led to perceived futility of the physician/student’s efforts.

*“I noted that over time*,* especially as patients gave me more pushback*,* I started to lose some empathy. While I inherently understood that patients may present as irritable for a variety of reasons (e.g. sickness*,* vulnerability)*,* it started to affect me more on a personal level…”* (P3).

In some cases, the depersonalisation or dehumanisation acted as an (at least in part) intentional measure, intended to protect the caring and compassionate participant from further emotional injury. In other cases, it was presented as a subconscious response mechanism to challenging patient behaviours which, at times, was only noted in retrospect.

“I don’t know what’s happening with my emotions but I realized today I no longer care about patients. Med school ripped pretty much all the empathy I had out of me and crushed any joy I found in medicine. I was looking over a chart today for a patient and just kept reading more bad decisions more bulls*** this person has done to themselves and all I could hear is blah blah who the f*** cares.” (Reddit post).

The other prominent struggle participants described was a lack of solidarity and community with other healthcare workers, whether physicians, other trainees, or other healthcare providers (i.e. nurses). Participants described a lack of support from those around them in the professional sphere, replaced instead with discrimination, toxic behaviours, lack of shared worldviews or values, and hierarchy rooted in injustice.

### Realised Emotions

As they progressed through their training, participants noted the development of certain emotions encouraging the loss of compassion and rise of cynicism, although the specific emotions and order arising differed by individual.

By and large, participants noted a realised helplessness, powerlessness, and loss of autonomy, especially as early medical students witnessing the traumas and culture of medicine, often for the first time. They noted repeated exposure to suffering without sufficient time to process or heal. Extreme hours and workload compounded physical exhaustion and mental exhaustion from the demands of the job:

They also noted a rigid hierarchy and fear of repercussions guiding their actions and decisions, in the face of secondary trauma and workload:

*“Easy to call us cowards like we could easily change our situation. There is a tremendous imbalance of power*,* by design*,* that does not favor medical students and residents….those in power will always use their discretion to threaten our livelihood against us; it’s not easy to advocate for work-life balance when said advocacy can be labeled as “unprofessional” and grounds for dismissal when you have 300k of debt.”* (Reddit post).

Fear of repercussions, workload, and feelings of powerlessness led to a lack of personal time and self-care. This included healing from the traumas and struggles encountered, as well as a deterioration of important relationships and support networks. A lack of space or recognition for grief and emotions intrinsic to medicine was also noted, at times due to time constraints and work pressures:

*“You think [patient is] doing OK and then all of a sudden they end up in the ICU …and then she died. And there wasn’t any emotional support or debriefing provided when she died. She just wasn’t on the list one day. And I asked my attending. And he was like*,* oh*,* yeah*,* she died. And then we just moved on to the next thing…you were given about 5 seconds to process it and move on like yes the patient died. Now we have to go see someone else”* (P4).

*“We are not fine. The innumerable traumas and suffering we see on a daily basis that the public does not understand…I hate being the one that has to crush that last ounce of hope or break catastrophic news.”* (Reddit Post).

Participants reported a constellation of emotions which began to develop as a response: bitterness, dread, resentment, disillusionment, regret, frustration, and anger. Compassion was noted by participants to be a finite commodity, one which requires effort that a tired trainee can’t give.

*“I think of your empathy*,* your compassion*,* you kind of have a bank*,* right…again we’re all human. There are limits to how much of any one emotion we can give to anybody*,* right?”* (P7).

In the face of challenging emotions and limited support, participants reported feeling trapped in medicine, fearing for job stability and financial stability, while weighing debt and lack of transferable skills. A number of participants also voiced frustrations with the lack of improvement in the medical system and workload, lack of perceived results of efforts, and perceived stagnation in their life stage. Some voiced hope of improvement down the road, while others voiced longer-lasting grief for lost dreams and pre-medicine lives.

*“Having no control over where I will be placed for rotations or residency is contributing to my feelings of despair and doubt*,* as I desperately want to be near my partner and other support systems. If I were not in debt I would very likely drop out now and work a more “normal” job”* (Reddit Post).

*“One of the most tiring things about this long*,* long career is feeling like your stuck at “student” for half your life. I’m so fed up of being in school all my life. I thought by now in my 30s I would be at the stage where I can go to work*,* come from work and enjoy the fruits of my labor. But here I am still studying for exams and answering to teachers.”* (Reddit Post).

Burnout was mentioned a number of times, a combination of all of these factors, as well as a conditioning to say “yes” and overextend oneself.

### Coping Mechanism

The loss of compassion and empathy and rise of cynicism was largely noted by participants to be a coping mechanism to trauma, stress, and demands of the job, which developed over time. Participants recognised that maintaining pre-medicine or early-medicine levels of compassion, etc., were simply not sustainable and would lead to burnout, if it had not already:

*“I asked one of the most senior people on the job how he does it every day. The answer you just need to become numb. I guess when you start to feel the suffering again it becomes unbearable.”* (Reddit Post).

*“I think over time there is a*,* you know*,* there’s a little bit of desensitization to things*,* right? Like you deal with different difficult things every day*,* right? I think you know*,* as human beings we adapt to any situation…”* (P7).

These transitions were noted not just with patients, but with colleagues, family, trainees, and even the participant themself. Medicine was noted to change its practitioners, forcing them towards undesirable qualities in order to cope with daily realities.

*“I just don’t think you understand how much you carry home with you and what weighs on you. And you understand that you take certain things home with you in that day. But I don’t think I really understood how much certain things will haunt you years after the fact. But there’s certain things that you just viscerally remember.”* (P5).

The COVID-19 pandemic was commonly cited as an example of severe secondary stress for which there was minimal support and altered worldviews and mindsets; however, other examples, such as a specific patient’s case or ongoing challenging situations without sufficient debrief or healing between, were also provided.

*“And I have seen it change [colleague]*,* when you put people in those inhumane situations and you expose people to secondary trauma day after day.”* (P4)*“I didn’t want to be the type of doctor that referred to people as diseases*,* like please go see the pneumonia in bed 3… people were doing that because they were being asked to see too many patients in too short a time…that was their coping mechanism…you couldn’t get to see people fast enough. There were too many people. You were too tired. You were always worried about making a mistake because you’d been up for 30 hours.”* (P4).

Many participants expressed concrete plans to improve their future and place boundaries on their practice to make it more sustainable, which often involved cutting back on work:

*“But I know that when I’m all done*,* and I can choose to do part time surgery or at least only elective surgery*,* it will all be ok.”* (Reddit Post).

However, the loss of compassion and empathy and rise of cynicism were noted to be an expected and anticipated process during medical training, difficult to avoid despite a trainee’s best efforts. Some participants expressed awareness when they were spiralling into an undesirable mindset, but lacked the time and energy to focus on themselves.

*“I see compassion as important*,* but I think there’s also a thing of having like*,* too much compassion…[I have to find] that balance between being compassionate but being able to go home and return to my regular life”* (P2).

## Discussion

Previous studies have demonstrated the loss of compassion [9] and rise of cynicism [6] in medical learners. As noted also in our study, this evolution becomes more profound over time [19]. We describe here a four-step process through which this evolution happens: a real-world awakening to the realities of the medical field, interpersonal struggles compounded by lack of support, realised emotions in response to these realities and struggles, and a coping mechanism manifesting as the loss of compassion and empathy and rise of cynicism.

Previous work in medical education (conducted, in fact, using social media posts as data sources) has noted an association between behaviours reinforcing hierarchy, long work hours, lack of support, and medical culture with the development of cynicism in learners [17]. Our four-milestone framework offers an explanation as to how these factors come together. Along the same lines, the loss of empathy is associated with (or triggered by) stressful work environments, pressures on performance, desensitisation, and burnout [9], the last of which was a common endpoint described by our participants. Recall that compassion (and empathy) are limited commodities which require effort and energy – the learner or physician struggling with burnout already faces a lack of energy to perform their expected tasks. Along these lines, the use of loss of compassion and empathy/rise of cynicism may even be seen as directly related to burnout – compassion and empathy require effort, which is already a crisis point for a physician experience burnout. Simultaneously, cynicism allows a physician experiencing burnout to separate from the encounter or stressor at hand.

Thus, in this study, we ultimately suggest that the two processes (loss of compassion and empathy/rise of cynicism) are synchronous coping mechanisms – whether conscious or unconscious (participants describe both) – to the daily realities of medical practice. Despite the nearly ubiquitous nature of this sentiment across all data sources, a number of participants noted feeling alone and ashamed in their feelings. Such feelings of shame, however, do not appear to be warranted. Our four-step model suggests a several potential points for future interventions:

- More accurate education of pre-medical students as to the realities of the field.
- More candid orientation for early medical students as to what they can expect (and moving this into the formal curriculum).
- Ensuring de-escalation and conflict training is included in communication education.
- Revising the current support and mentorship structures.
- Teaching alternative, healthier coping mechanisms for dealing with the realities of the medical field.
- Ongoing support and intervention opportunities for faculty, as teachers of learners, but also as physicians who may benefit from reassessment of coping mechanisms learnt as a survival mechanism.

Granted, this study is primarily hypothesis-generating, and does have several limitations. While Reddit does not mandate location identification, the wording and content of posts suggested a primarily North American audience; additionally, despite second-phase participants being invited from across North America, ours were exclusively from Canada. Previous literature suggests that the loss of empathy could, indeed, be driven by geographical location [20], and our process may not translate into, for example, Asian or European settings, given differences in training and practice demands. Furthermore, our study was cross-sectional, capturing perspectives at a variety of stages in a single point in time. A longitudinal study following a diverse cohort of trainees could provide additional information and confirmation of this study’s findings. Additionally, unlike other studies in the area, we are relying on participants’ own perceptions at a single point in time, rather than validated scales or other objective ways of measuring compassion, empathy, and cynicism. However, when considering burnout, job satisfaction, and retention in the field, the perception of the individual trainee is of paramount importance.

In any case, it is clear that there is a process with milestones for the loss of compassion and empathy and rise of cynicism – one which needs to be interrupted. While curricula exist to build and rebuild compassion [21], we have highlighted here a number of places the focus can be on *maintaining* compassion and empathy and turning medicine into a sustainable career – and not one where trainees focus on a future goal of cutting back and leaving their careers as soon as feasible. 

## Data Availability

The data which support findings are available from the authors upon reasonable request.
